# Treating Parvovirus Triggered Refractory Hemolytic Anemia with Rituximab in Renal Transplant Recipients – A Report of Two Cases

**DOI:** 10.4103/ijot.ijot_34_22

**Published:** 2023-01-01

**Authors:** Chilaka Rajesh, Utkarsh Mishra, Anna Valsan, Elenjickal Elias John, Jeethu Joseph Eapen, Athul Thomas, Sabina Yusuf, Suceena Alexander, Vinoi George David, Santosh Varughese

**Affiliations:** Department of Nephrology, Christian Medical College, Vellore, Tamil Nadu, India

**Keywords:** Parvovirus B19, renal transplant, rituximab

## Abstract

Parvovirus B19 is a small (26 nm), nonenveloped, single-stranded DNA (5.6-kb) virus. The only known host for parvovirus B19 is humans. Parvovirus B19 is directly cytotoxic to erythroid precursor cells of the colony- and burst-forming units. Human parvovirus B19 is the etiologic agent of erythema infectiosum and chronic pure red cell aplasia in immunocompromised individuals. Acute parvovirus B19 infection should be suspected in immunocompromised patients, who present with reticulocytopenic hemolytic anemia and thrombocytopenia. Intravenous immunoglobulin (IVIg) is the standard treatment for parvovirus-induced cytopenias. We report two cases of postrenal transplant who presented with reticulocytopenic anemia and were found to have parvovirus infection. They did not respond to conventional treatment with intravenous gamma globulin. Both patients were treated with rituximab with which they had improvement in clinical and hematological parameters. There was no previous documentation of using rituximab in the treatment of parvovirus-triggered autoimmune hemolytic anemia postrenal transplant patients. This article illustrates how rituximab will be helpful in this setting, of course, it is a new thought but requires further studies and validation.

## Introduction

Human parvovirus B19 (HPV-B19) is the etiologic agent of erythema infectiosum and causes transient aplastic crises. In immunocompromised individuals, persistent parvovirus B19 viremia presents as chronic pure red cell aplasia (PRCA).^[1]^ HPV-B19 rarely has been implicated as a cause of autoimmune hemolytic anemia.^[2]^ The cellular receptor of HPV-B19 is the blood Group P antigen, which explains the viral tropism for erythroid precursors.^[3]^

We report two cases of renal allograft recipients with parvovirus-related PRCA, who did not respond to conventional intravenous gamma globulin treatment. They were treated with rituximab following there was an improvement in clinical and hematological parameters.

## Case Reports

### Case 1

A 27-year-old male patient, a case of renal allograft recipient in January 2017, ABO compatible with pretransplant negative cross match and received basiliximab induction. His initial maintenance immune suppression was prednisolone and tacrolimus. Mycophenolate was not started in view of persistent leukopenia. In the immediate posttransplant on day 10, he had acute antibody-mediated rejection and was treated with pulse methylprednisolone and plasmapheresis. One month of posttransplant, he had acute cellular rejection and received anti-thymocyte globulin for the same. His nadir creatinine reached to 1.8 mg/dl. Three months posttransplant, he presented with acute febrile illness with pancytopenia. On evaluation, he was found to have mean hemoglobin of (5.7 ± 7.2 g/dl), platelets (41,000 mm^3^ ± 70,000), lactate dehydrogenase (LDH) - 823, reticulocyte count - 0.08%, unconjugated hyperbilirubinemia, and positive direct Coomb’s test. Blood and urine cultures were sterile and imaging did not show any focus of infection. Tropical work up such as serology for dengue, leptospira, and scrub typhus was negative. CMV polymerase chain reaction (PCR) was negative.

**Figure 1:**
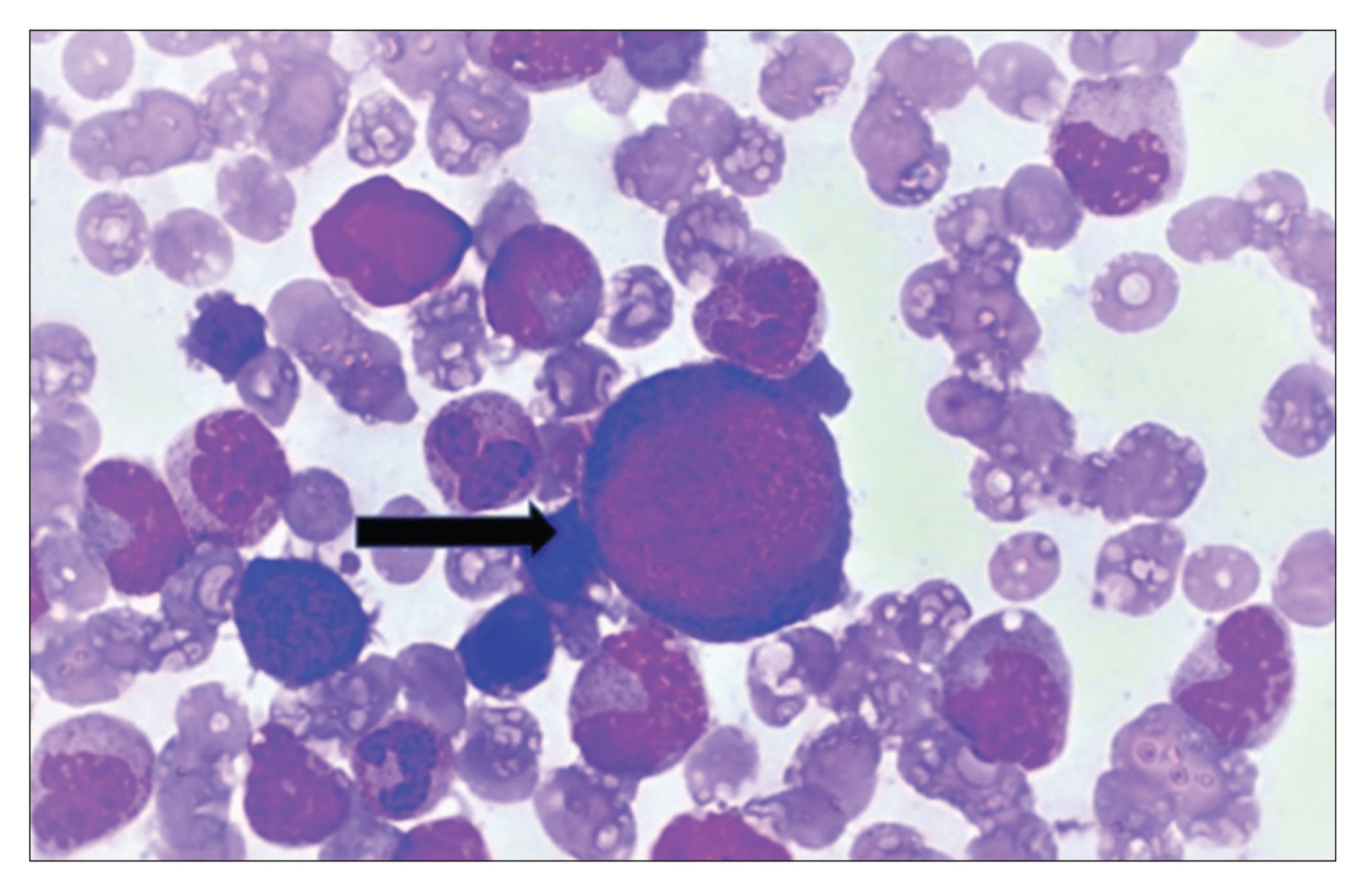
Bone marrow aspiration: Giant proerythroblast (black arrow) (MGG, ×100)

**Graph 1:**
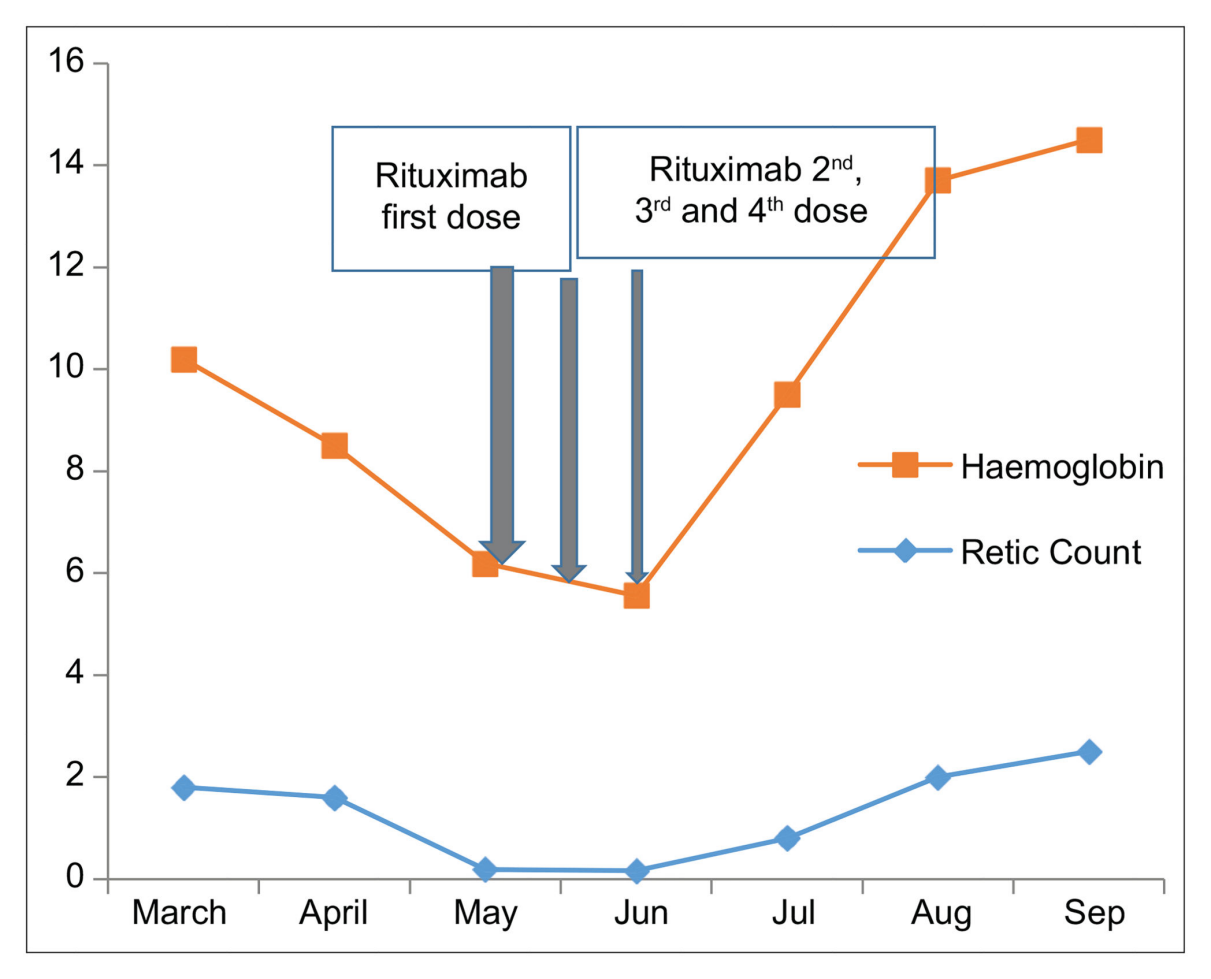
Case 1 - Hemoglobin and reticulocyte count trend

On further evaluation, parvovirus infection was confirmed by real-time qualitative PCR and bone marrow studies showed PRCA [Figure 1]. He was managed with IV immunoglobulins (IVIg) at 400 mg/kg/day for 5 days. He did not show any response to IVIg and the same dose was repeated after 1 week. The hemoglobin was 8 g/dl and the platelet count was 70,000 mm^3^ after IVIg therapy. He was given four doses (weekly once) of rituximab at 375 mg/m^2^ following which there was an improvement in cytopenias with maximum hemoglobin of 11.6 g/dl and platelets count to 96,000/mm^3^. He had stable graft function during this period [Graph 1].

### Case 2

**Figure 2:**
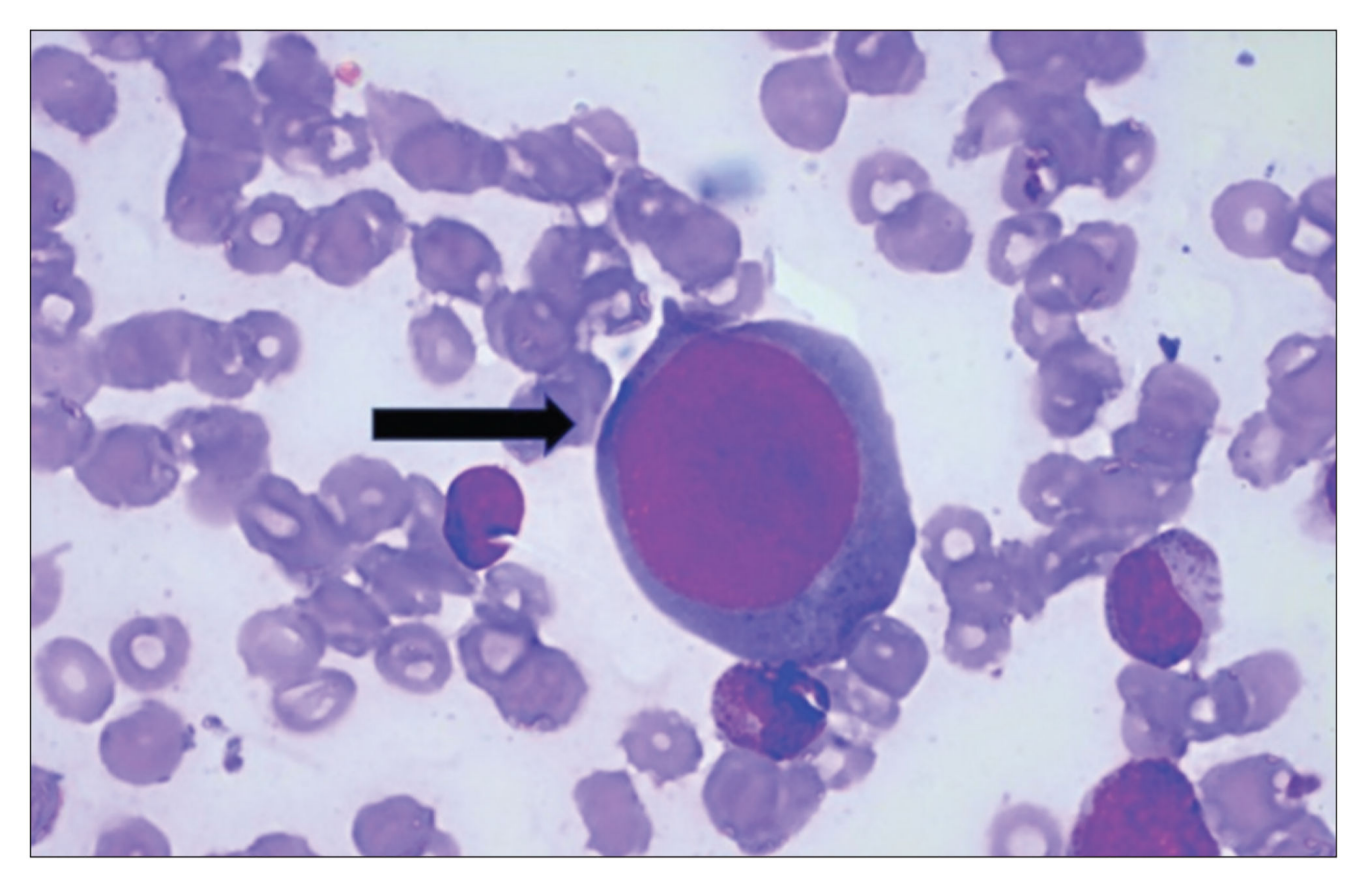
Bone marrow aspiration: Giant proerythroblast with intranuclear inclusions (black arrow) (MGG, ×100)

**Graph 2:**
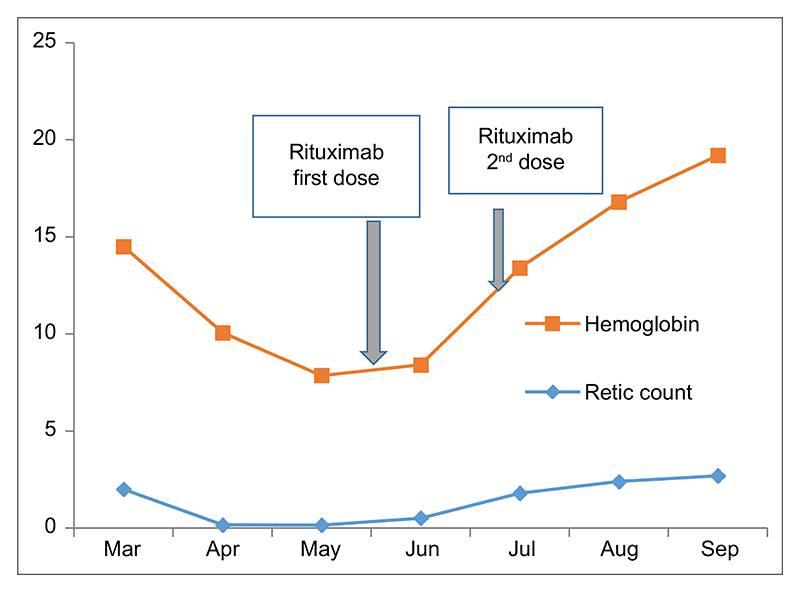
Case 2 - Hemoglobin and reticulocyte count trend

A 31-year-old male patient, case of renal allograft recipient in March 2020, ABO compatible with pretransplant positive crossmatch and received desensitization as per institute protocol with injection rituximab 500 mg 1 week before transplant and two sessions of plasmapheresis. Antithymocyte globulin induction was given. He received maintenance immunosuppression with prednisolone and tacrolimus. Mycophenolate was stopped in view of persistent leucopenia. He had no rejections. During posttransplant follow-up, he was detected to have persistent pancytopenia. His investigations were hemoglobin (5.7 ± 7.6 g/dl), reticulocyte count 0.56%, LDH - 547 and positive direct Coomb’s test. On evaluation, parvovirus serology was positive and bone marrow studies showed PRCA [Figure 2]. He was given IVIg at 400 mg/kg/day for 5 days, twice, 2 weeks apart with which he had no improvement. He was treated with two doses (weekly once) of rituximab at 375 mg/m^2^. Subsequently, cytopenias were improved with hemoglobin of 12.6 g/dl. He had has stable graft function during this period [Graph 2]. The summary of both the cases is shown in Table 1.

## Discussion

Infection is one of the important causes of autoimmune hemolytic anemia (AIHA). Specific infectious agents that may trigger AIHA include *Mycoplasma pneumoniae*,^[4]^ Epstein–Bar Virus (EBV),^[5]^ measles, varicella, adenovirus, mumps, and rubella. Most of these infections are associated with cold agglutinins due to IgM autoantibodies with specificity for the I/i polysaccharide antigen system on red cells, although reactivity with the *P* polysaccharide antigen has been reported.^[6]^

Parvovirus-B19 not only causes a transient aplastic crisis in patients with reduced red cell survival, but it also triggers AIHA. Five cases of AIHA due to acute HPV-B19 infection in healthy children were reported.^[2]^ Few cases of HPV-B19-induced AIHA associated with hemophagocytic syndrome have also been published.^[7–9]^

The frequency of parvovirus B19 viremia among immunocompromised patients may be underestimated. As an example, in a study of 60 renal transplant patients who underwent nucleic acid testing for various opportunistic viruses within the 1^st^ year after renal transplant, 10% had parvovirus B19 viremia, compared with 13 and 12% with CMV and EBV reactivation. The majority of the cases were reactivation as opposed to primary infection, and anemia was not more frequent among the patients with parvovirus B19 viremia.^[10]^

In a retrospective review of 98 transplant recipients who developed posttransplant parvovirus B19 infection, the median time to onset of disease was 7 weeks after transplantation. Anemia, leukopenia, and thrombocytopenia occurred in 99%, 38%, and 21% of patients, respectively.^[11]^

HPV-B19 is the etiologic agent of erythema infectiosum and chronic PRCA in immunocompromised individuals. Acute parvovirus B19 infection should be suspected in immunocompromised patients, who present with reticulocytopenic hemolytic anemia and thrombocytopenia. Parvovirus B19 virus infection usually is asymptomatic or mildly symptomatic with self-limited anemia in people without hemolytic diseases. Because of the inability or decreased ability to mount an immune response to clear viremia, chronic or reactivated parvovirus B19 infection can occur in immunosuppressed individuals. This can lead to hypoplasia or aplasia of the erythroid cells and precursors and severe acute or chronic anemia, which can be life-threatening.

PRCA is characterized by anemia, reticulocytopenia, and erythroblastopenia in the bone marrow. It may be congenital or acquired. Acquired causes of PRCA include various infections such as HPV-B19, hematological malignancies such as chronic lymphocytic leukemia (CLL), chronic hemolytic anemias, autoimmune diseases, and various drugs. The pathogenesis of PRCA is not clearly understood; the mechanism is believed to be mediated by humoral antibody or natural killer (NK) or T-cell-mediated damage to precursors. Humoral immunity-mediated damage includes complement-mediated lysis, formation of antibody-EPO complex, EPO receptor blocking antibody, and increased production of autoantibodies due to T-cell dysfunction, or blockade of burst-forming units-erythroid (BFU-E) differentiation.^[12,13]^ NK/T-cell-mediated damage includes cytolysis against bone marrow proerythroblasts or autoantibody formation against bone marrow proerythroblasts which is directed toward the Fc receptor.

Humoral immunity-mediated damage is seen in systemic lupus erythematosus, rheumatoid arthritis, lymphoma, and thymoma, whereas NK/T-cell-mediated damage occurs in CLL, large granular lymphocytic leukemia, and lymphoma. The formation of anti-EPO antibodies and inhibition of EPO-dependent cell lines are important mechanisms of PRCA in SLE patients with AIHA.

PRCA associated with AIHA is caused by humoral as well as cytotoxic immunity. An interesting postulation is the coexistence of two different autoantibodies to the erythroid series, and the clinical manifestation may then be determined by a dominant antibody effect. A cytotoxic effect is mediated by both peripheral blood and bone marrow mononuclear cells.^[14]^ Taniguchi *et al*. have demonstrated two distinct immunological mechanisms in the pathogenesis of PRCA in their study: One is T-lymphocyte-mediated and the other being complement-dependent IgG-mediated suppression of erythroid progenitors. Both T-lymphocyte and autoantibody-mediated inhibition of erythropoiesis occur at the level of colony-forming units-erythroid and BFU-E.

In typical AIHA cases, the reticulocyte count is elevated, while our patients had profound reticulocytopenia, a well-known feature of acute parvovirus B19 infection. The bone marrow showed erythroid hypoplasia along with the presence of giant pronormoblasts which are typical findings of parvovirus B19 infection. Parvovirus B19 infection in our cases was confirmed with real-time PCR Thus, in all likelihood, parvovirus B19 infection triggered the hemolysis in our patients. Both patients showed PRCA on bone marrow.

In the setting of chronic infection with anemia in immunosuppressed patients, treatment with IVIG and reduction of immunosuppression is suggested as per the American Society of Transplantation for the treatment of symptomatic parvovirus B19 infection in solid organ transplant recipients. In patients with solid organ transplants or other non-HIV causes of immunosuppression, 400 mg/kg/day of IVIG for 5 consecutive days is recommended.

Both patients were treated with immunoglobulin 400 mg/kg body weight for 5 days, but there was no response. Since rituximab was used as off labeled drug in cases of refractory AIHA and was used in the treatment of EBV,^[15]^ we thought to give rituximab at 375 mg/m^2^ dose based on the fact that parovovirus-triggered AIHA in these two cases and immune-mediated etiopathogenesis was described in this setting. Both the patient showed clinical and hematological improvement with stable graft function which was confirmed by graft kidney biopsy since many case reports of rituximab causing reactivation of parvovirus b19 have been reported it is critical to use this drug in all cases and tried only in selected cases where IVIg has failed to show response with patients positive for parvovirus B 19 with AIHA.

## conclusion

The use of rituximab in parvovirus-triggered hemolytic anemia in postrenal transplant patients requires further studies as there was no documentation in the literature. Using intense immunosuppression is always double-edged sword in transplant patients as it may reactivate other infections. Clinicians can consider using rituximab after assessing risk–benefit ratio when they encounter renal transplant patients with parvovirus-triggered refractory reticulocytopenic AIHA. More randomized control trials are needed to validate the use of rituximab in the treatment of parvovirus B 19-triggered AIHA.

## Figures and Tables

**Table 1:** Summary of clinical and treatment details of the patients

Age/sex	Year of transplant	ABO compatibility	Pre-Tx crossmatch	Induction		Maintenance	Rejections
27/male	January 2017	ABO compatible	Negative	Basiliximab		Predisolone Tacrolimus MMF	ABMR - required TPE ACR - required ATG
31/male	March 2020	ABO compatible	Positive	ATG		Predisolone Tacrolimus MMF	Nil
**Timing** **of**	**Presentation**	**Investigations**	**Parvovirus**	**Graft**	**Bone**	**Respsone** **to** **IVIg** **400**	**Response** **to** **rituximab**
**parvo** **infection**			**PCR** **qualitative**	**function**	**marrow**	**mg/kg/day** **×5** **days**	**375** **mg/m^2^** **weekly**
3 months post-Tx	Acute febrile illness with pancytopenia	Hb -5.7±7.2 Retics - 0.08% LDH - 823 DCT -positive	Detected	Stable	PRCA	No	Yes - four doses
5 months post-Tx	Refractory anaemia	Hb -5.7±7.6 Retics - 0.56% LDH - 547 DCT - positive	Detected	Stable	PRCA	No	Yes - two doses

Hb: Hemoglobin, LDH: Lactate dehydrogenase, DCT: Direct coombs test, ATG: Anti thymocyte globulin, MMF: Mycophenolate mofetil, ABMR: Antibody-mediated rejection, ACR: Antithymocyte globulin, PCR: Polymerase chain reaction, IVIg: Intravenous gamma globulin, PRCA: Pure red cell aplasia
